# Letter from New York

**Published:** 1889-07

**Authors:** 


*To the Editors Atlanta Medical and Surgical Journal:*

At a recent meeting of the societies on Public Health of the Academy of Medicine, Dr. W. E. Forrest spoke on the subject of the cost of yellow fever epidemics. The doctor was present during last year's epidemic in Alabama, and has made a special study of the subject. He gave statistics gathered from a large number of outbreaks of this disease, and said that the epidemic in 1878 cost the country over $200,000,000.

The fever is, he believes, essentially an urban disease and can be easily stamped out. It is not contagious from one patient to another, but it is infectious in a particular locality, in which the germs find a suitable place to multiply. He recommended the removal of those threatened with the fever to distant camps, where there was little liability of contracting it. During the first three days of the disease, Dr. Forrest stated that the patient might be moved about with impunity, but when the period of calm arrives, careful watching was necessary. During this period the patient feels well and often asks to get up and eat; allowing him to do either will often bring on a fatal result, and even changing the clothes may kill him at this time. During the evening he referred to the late Prof. Proctor who was removed from a New York hotel, suffering from yellow fever and died soon after. This the doctor said, was done during the period of calm, and the man died of exposure.

He believes when the disease breaks out, the government should immediately take charge of the locality, remove the residents and quarantine the place. Referring to New York, he did not believe it would be possible to introduce the disease here, owing to the streets being paved and the perfection of the sewerage system. The germs he said, must have a chance to grow before becoming dangerous, and in this city they wouldn't get that chance.

It was resolved to refer the question of supporting the bill, which General Wheeler, of Alabama, introduced in Congress, to the consideration of the Academy at its next regular session. This bill embodies the views of yellow fever experts upon the national management of the disease.

It is expected that the Academy will soon have a new building, as already a piece of ground has been selected in 43d Street, west of 5th Avenue.

A new method of managing cerebral hemorrhage, when the patient can be seen early, has been devised by Dr. Andrew H. Smith of this city, with a view to limiting as much as possible the amount of blood effused. He believes the rupture occurs in most of these cases, not in the continuity of an artery, but in the wall of a small aneurism upon the vessel. Nature's usual methods for arresting bleeding do not apply here. There can be no contraction of the circular muscular fibres, as occurs in ordinary wounds, for the wall of the aneurism is destitute of this tissue; neither will there be any longitudinal retraction of the artery within its sheath, as the vessel is not completely divided. The brain tissue is too soft to prevent the outflow of blood into its substance, and the hemorrhage is never profuse enough to stop, from a weakened heart's action. All these means failing, Dr. Smith says that when the bleeding has compressed the brain to its utmost, and no more room is left within the cranium for the blood to be effused into, the bleeding then necessarily stops. Dr. Smith, therefore advises in cases when we believe the bleeding to be still going on, to bring about this pressure as soon as possible, and it is better he says, to do this by blood within the vessels than by blood extravasated into the cranial cavity. He argues that the more blood there is in the inter-cranial vessels, the less room there will be for blood outside of them, and consequently the head should be lowered and the cerebral congestion increased by nitrate of amyl, which drug would also tend to lower arterial tension. He is opposed to the use of ergot in this condition on the following grounds: The diseased condition of the vessels leaves no muscular fibre for it to act upon, and even any remaining would most likely be on the distal side of the opening, in which case the hemorrhage would be increased. Ergot also raises arterial tension and would consequently drive the blood out of the opening with increased force. Dr. Smith also objects to purging and bloodletting, which he says only diminish the total amount of blood in the body. He condemns the practice of elevating the head because it lessens the quantity of blood in the cranium. The application of cold to the head he considers a proper procedure, not because it may contract the vessels, but because reducing the temperature favors the coagulation of blood.

Since the writing of my last letter, Dr. John C. Dalton, one of our most distinguished physiologists, has died. He had held the position of professor in three medical colleges, before beginning his duties at the College of Physicians and Surgeons in this city. Dr. Dalton became a physiologist early in his professional life, and it is stated that he was the first in this country to practice vivisection as a means of teaching physiology. Those who attended his lectures will remember him as an unusually interesting and instructive speaker, whose style was so terse and simple that any student could follow him. Besides his work on Human Physiology, which is used very largely as a text book in many institutions, he was the author of numerous other valuable publications.

Dr. Alfred L. Loomis was a few days ago examined as a witness in a suit brought against him by the wife of the late Dr. M. W. Miller. This lady claims that after the death of her husband, who was a director of the Loomis Laboratory, his books, specimens and instruments were taken by Dr. Loomis. She also states that the paper which Dr. Loomis published in the Medical Journal, entitled "Cardiac Changes in Chronic Bright's Disease" was written by her husband. Dr. Loomis denies all these charges, and states that he only owed Dr. Miller a portion of his salary, which the widow was welcome to.

A. F.

New York, 1889.

